# Optical Properties of Oxidized Plasma-Polymerized Organosilicones and Their Correlation with Mechanical and Chemical Parameters

**DOI:** 10.3390/ma12030539

**Published:** 2019-02-12

**Authors:** Bozena Cechalova, Martin Branecky, Petr Klapetek, Vladimir Cech

**Affiliations:** 1 CEITEC, Brno University of Technology, Purkynova 123, 612 00 Brno, Czech Republic; bozena.cechalova@ceitec.vutbr.cz; 2 Institute of Materials Chemistry, Faculty of Chemistry, Brno University of Technology, Purkynova 118, 612 00 Brno, Czech Republic; xcbranecky@fch.vut.cz; 3 Czech Metrology Institute, Okruzni 31, 638 00 Brno, Czech Republic; pklapetek@cmi.cz

**Keywords:** plasma polymerization, thin films, spectroscopic ellipsometry, optical properties, chemical properties

## Abstract

Pure tetravinylsilane and its oxygen mixture were used to deposit oxidized plasma polymer films at various effective power (0.1–10 W) and various oxygen fractions (0–0.71) using RF pulsed plasma. The optical properties (refractive index, extinction coefficient, band gap) of the deposited films were investigated by spectroscopic ellipsometry (230–830 nm) using an optical model and Tauc‒Lorentz parametrization. Analyses of chemical and mechanical properties of films allowed for the interpretation of changes in optical properties with deposition conditions. The refractive index was revealed to increase with enhanced effective power due to the increased crosslinking of the plasma polymer network but decreased when increasing the oxygen fraction due to the decrease of polymer crosslinking as the number of carbon bonds in the plasma polymer network was eliminated. A very strong positive correlation was found between the Young’s modulus and the refractive index for oxidized plasma polymer films. The optical properties of films correlated with their chemical properties for the specific deposition conditions used in this study. The band gap (1.9–2.9 eV) was assumed to be widened due to the increased concentration of vinyl groups in oxidized plasma polymer films.

## 1. Introduction

Optical coatings are functional materials in the form of thin films that are deposited on optical components to improve and control their optical phenomena in optical and optoelectronic devices. The coatings are applied as a single layer, multilayer, or gradient film, where the optical properties (refractive index, extinction coefficient) are constant, stepwise changing, or continuously varying across the film, respectively. The optical properties are inevitably related to other functional characteristics such as mechanical properties, adhesion, or thermal stability. Such optical coatings can be used as reflective, antireflective, or transmissive coatings, transparent electrodes, and optical filters in healthcare, military, electronics, transportation, or even construction [1].

Optical coatings are mostly deposited by physical vapor deposition (PVD), e.g., magnetron sputtering [1,2], but chemical vapor deposition (CVD) offers a wider range of technological tools to control not only optical but also other physical, chemical, and surface properties of deposited micro- or nanostructures, since the total coating thickness can range from several nanometers to several micrometers. Among the CVD techniques, plasma-enhanced CVD (PECVD) with low-pressure non-thermal plasma appears to be a convenient process of synthesizing very complex optical coatings in one deposition [3]. If organic or organosilicon precursors are used at low plasma densities, polymer-like coatings, referred to as plasma polymers, and deposition techniques such as plasma polymerization may be applied [4]. The precursor (monomer) molecules are ionized and dissociated in plasma (glow discharge), resulting in the deposition of plasma polymer on substrates of various shapes at low substrate temperatures (<100 °C). Plasma polymer films are amorphous materials with a high concentration of hydrogen atoms (40–60 at %) [5].

The mechanical properties (Young’s modulus, hardness) of plasma polymer films depend on the chemical structure of the plasma‒polymer network and its crosslinking. It was found that the mechanical properties of plasma-polymerized tetravinylsilane films can be controlled by the effective power using RF (radio frequency) pulsed plasma [6]. Differences in the plasma polymer network affected by the effective power were introduced by Cech et al. [7]. Incorporation of oxygen atoms into the plasma polymer network led to the formation of stronger chemical species in the backbone chain and side polar groups (hydroxyl, carbonyl) that reduced crosslinking of the plasma polymer network [6]. This chemical modification influenced not only the mechanical properties of plasma polymer films but also their adhesion to the silicon dioxide surfaces [8] and their surface free energy (wettability) [9].

We focus on plasma polymer films of tetravinylsilane as this type of material allows us to control not only the degree of its organic/inorganic nature but also the level of network crosslinking of the plasma polymer in a relatively wide range depending on the deposition conditions, resulting in a high application potential of this material. Plasma-polymerized tetravinylsilane films can be applied as low-k dielectrics, gas barrier coatings, corrosion protection, antireflection coatings, optical filters, dielectric coatings, or compatible interlayers in hybrid materials like composites and nanocomposites [10].

This study focuses on the optical properties (refractive index, extinction coefficient, band gap) of plasma polymer films deposited from the pure tetravinylsilane monomer and its mixture with oxygen gas using RF pulsed plasma, which were characterized by spectroscopic ellipsometry. The optical properties were correlated with the Young’s modulus and chemical parameters of the corresponding plasma polymer films to understand how the chemical structure of plasma polymer network and its crosslinking determine the physical properties of deposited films.

## 2. Materials and Methods

### 2.1. Plasma Polymerization Technique

Plasma polymer films were deposited on infrared-transparent silicon wafers (100) (0.8 × 10 × 10 mm, ON Semiconductor, Roznov pod Radhostem, Czechia) using an RF (13.56 MHz) helical coupling system (RF glow discharge) operated in a pulsed regime [11]. Tetravinylsilane, Si(-CH=CH_2_)_4_ (TVS, Sigma Aldrich, Prague, Czechia), was used as the monomer. Argon gas was used to clean the plasma reactor and vacuum chambers. Oxygen gas was mixed with TVS monomer to deposit plasma polymer films with inbuilt oxygen atoms. The silicon wafer was pretreated with O_2_ plasma (5 sccm, 4 Pa, 25 W) for 10 min to improve film adhesion. The wafer was then stored in a load lock. Using a linear driver, the pretreated wafer was placed in the plasma zone after setting the deposition conditions and the plasma reached a steady state that was monitored by mass spectroscopy. Precursor molecules are fragmented during the plasma process, and plasma polymer formation is thought to be the result of the stepwise reactions (atomic processes) that predominate, as suggested by Yasuda [12]. The total flow rate (TVS + O_2_) of 0.55 sccm was constant and the ratio of oxygen to total flow rate, i.e., the oxygen fraction in TVS/O_2_ mixture, was set to 0.00, 0.10, 0.21, 0.33, 0.46, and 0.71. The corresponding process pressure was approximately 1.4 Pa. The effective power used was 0.1, 0.5, 2.5, 5.0, and 10 W at *t*_on_ = 1 ms (the time for which the plasma was switched on). Finally, the deposition chamber was purged with argon gas (10 sccm, 10 Pa) for 30 min and then flooded with air to atmospheric pressure. The efficiency of the plasma polymerization process can be evaluated based on the monomer consumption, which ranged from 2% to 48% depending on the deposition conditions. The aging of the deposited films can be observed as a result of post-deposition oxidation of the plasma polymer [13], and therefore the analyses were performed within one week after the film was deposited.

### 2.2. Thin Film Characterization

Elementary thin film composition was studied by conventional and resonant Rutherford Backscattering Spectrometry (RBS) and Elastic Recoil Detection Analysis (ERDA) methods using a Van de Graaf generator with a linear electrostatic accelerator. Infrared measurements in the wavenumber range from 400 to 4000 cm^−1^ were performed using a Nicolet Impact 400 Fourier transform infrared (FTIR) spectrophotometer (Thermo Electron Corporation, Madison, Wisconsin). Transmission spectra were obtained on films deposited on one-side polished crystalline silicon wafers. To remove the spectral features of silicon wafers, absorption subtraction technique was used and the background correction was applied before each measurement to avoid all environmental contributions. Spectral resolution was 2 cm^−1^. To reach a sufficient signal-to-noise ratio, 128 scans were recorded. Surface topography of plasma polymer films was observed by atomic force microscope, AFM (Accurex IIL, Topometrix, Austin, TX, USA) and the root-mean-square (RMS) roughness was determined from the surface profile. The ellipsometric spectra of the deposited films were recorded using a phase-modulated spectroscopic ellipsometer UVISEL (Jobin Yvon HORIBA, Chilly-Mazarin, France) ranging from 230 to 830 nm to characterize the film thickness and dispersion curves for the refractive index and the extinction coefficient. The film thickness of the observed films was approximately 1 μm.

### 2.3. Ellipsometric Spectra Analysis

Ellipsometric spectra were analyzed using a multilayered ambient/overlayer/film/substrate optical model, which was found to be convenient in describing the depth profile of the samples. The model consisted of a semi-infinite substrate (silicon wafer with a 4-nm-thick native silicon dioxide layer), plasma polymer film, and a relatively thin (~10 nm) overlayer. The introduction of the gradient interlayer between the plasma polymer film and the substrate proved to be superfluous in interpreting the data.

The dielectric function, *ε*, is a complex quantity with the real, *ε*_1_, and the imaginary part, *ε*_2_, given by *ε* = *ε*_1_ + *i ε*_2_, where the real and imaginary parts are given as

(1) $$\begin{array}{l} \varepsilon_{1}=n^{2}-k^{2}\\ \varepsilon_{2}=2nk, \end{array}$$

where *n* is the refractive index and *k* is the extinction coefficient. The dispersion dependence of the dielectric function (Equation (1)) was fitted using the five-parametric Tauc‒Lorentz formula (Equation (2)), which was derived for parameterization of the optoelectric response of amorphous dielectrics [14]. It is based on the Tauc expression for the imaginary part of the dielectric function above the band edge [15] and the classical Lorentz oscillator model. In the Tauc‒Lorentz expression, the imaginary part of the dielectric function *ε*_2_ (*E*) is parameterized by four parameters, which are the product of the oscillator amplitude and the Tauc constant *A_T_*, the broadening term Γ, the peak transition energy *A*_0_, and the optical band gap *E_g_*:

(2a) $$\varepsilon_{2}(E)=\{\begin{array}{ll} \left(\frac{A_{T}\Gamma {\left(E-E_{g}\right)}^{2}}{(E^{2}-E_{0}^{2})+\Gamma^{2}E^{2}}\right) & E>E_{g}\\ 0 & E\leq E_{g} \end{array}.$$

The real part of the dielectric function is obtained by Kramers‒Kronig integration:

(2b) $$\varepsilon_{1}(E)=\varepsilon_{1}^{\infty }+\frac{2}{\pi }P\int_{E_{g}}^{\infty }\frac{\xi \varepsilon_{2}\left(\xi \right)}{\xi^{2}-E^{2}}d\xi ,$$

where *P* stands for the Cauchy principal part of the integral and the additional fitting parameter *ε*_1_^∞^ was included. This parameterization best matches the fits of ellipsometric spectra as plasma polymer films can be considered amorphous dielectrics.

Ellipsometric spectra were recorded using *I_s_* and *I_c_* parameters that correspond to the amplitudes of photoelastically modulated light intensity and can easily be converted to classical ellipsometric parameters (*P*), using the following equations:

(3a) $$I_{s}=\sin2\psi \sin\Delta$$

(3b) $$I_{c}=\sin2\psi \cos\Delta ,$$

**Figure 1:**
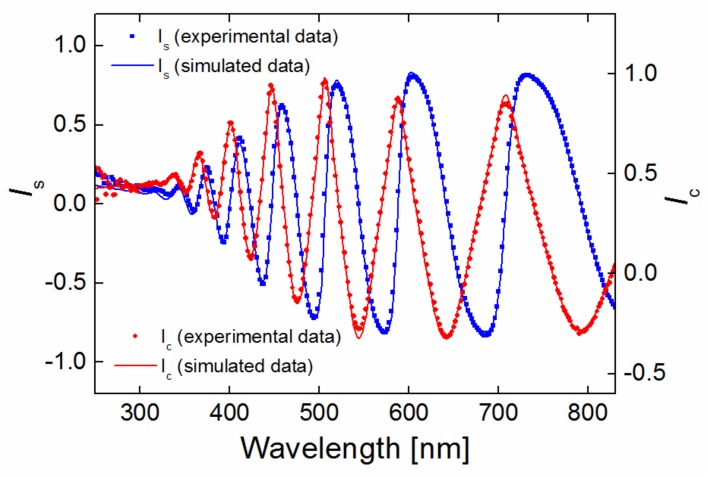
Experimental and simulated ellipsometric spectra.

The measured ellipsometric spectra (full symbol, Figure 1), *I_s_* (Equation (3a)) and *I_c_* (Equation (3b)), were fitted (solid line, Figure 1) by minimizing the squared difference between the experimental and simulated values of the parameters, expressed by the commonly used parameter χ^2^, given as follows [16]:

(4) $$\chi^{2}=\frac{1}{N_{dp}-N_{fp}-1}\sum \frac{{\left(\rho_{\exp}(\lambda_{i})-\rho_{calc}(\lambda_{i},z)\right)}^{2}}{\delta \rho {\left(\lambda_{i}\right)}^{2}},$$

where *N_dp_* is the total number of data points, *N_fp_* is the number of fitted parameters, *ρ_exp_* (*λ_i_*) is the experimental data at a given wavelength, *ρ_exp_* (*λ_i_*, *z*) is the calculated quantity associated with the experimental data and for the parameter vector *z* (with *N_fp_* elements), and *δρ* (*λ_i_*) is the error associated with each of the experimental data points. In our case, the average value of χ^2^ (Equation (4)) was approximately 10, which is interpreted as good accuracy for 1-µm-thick films.

## 3. Results

### 3.1. Chemical Analysis of Thin Films

**Figure 2:**
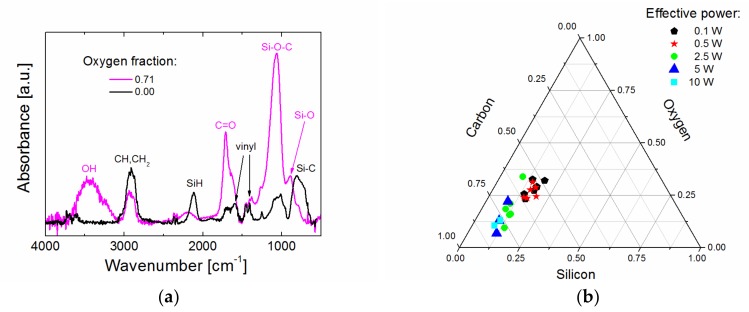
Chemical analysis of thin films: (**a**) infrared spectra for films deposited from pure TVS monomer and TVS/O_2_ mixture at an oxygen fraction of 0.71 and 2.5 W; (**b**) ternary diagram of elemental composition for films deposited at different effective power.

The chemical analysis (RBS, ERDA, FTIR) of plasma polymer films allowed us to formulate the conception of a plasma polymer as hydrogenated carbon silicon network with side vinyl groups (0.1 W), which was transformed into a mostly carbon network (10 W) as the organic/inorganic nature of the plasma polymer, expressed by the ratio of carbon to silicon, increased from about 3 to almost 8 with enhanced power. By increasing the oxygen fraction, the oxygen atoms were partly incorporated into the plasma polymer network, forming Si‒O‒C bonding species due to high-affinity oxygen capture by silicon atoms and partly forming side hydroxyl and carbonyl groups, which only slightly eliminated vinyl species (Figure 2a). Thus, deposition conditions have enabled the elementary composition and chemical structure of plasma polymer films to be controlled. The silicon concentration (Si) was approximately 5‒7 at % for all films and the carbon concentration (C) was approximately 35‒39 at % for films deposited at higher power (10 W), but decreased to 22 at % with increased oxygen concentration (O) of 3‒19 at % for films deposited at lower power (0.1 W). The concentration of hydrogen (H) was approximately 50‒55 at % for all films except for the highest oxygen fraction, at which the concentration dropped to 35 at %. The elemental (Si, C, and O) composition of plasma polymer films is plotted in a ternary diagram (Figure 2b), which indicates that the silicon concentration was almost invariant, but the carbon concentration was reduced at the expense of the oxygen concentration. Data are differentiated according to the effective power. Details of chemical analysis can be found in previous studies [17,18].

### 3.2. Deposition Rate

**Figure 3:**
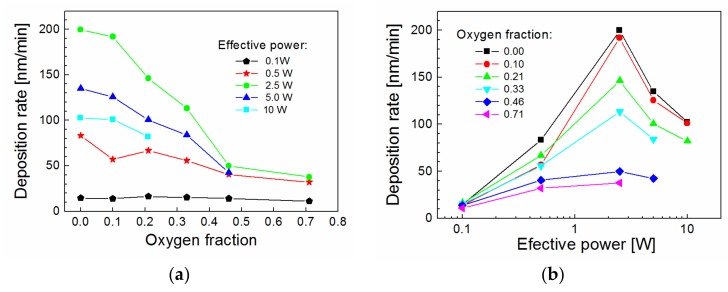
Deposition rate as a function of (**a**) oxygen fraction for different effective power and (**b**) effective power for different oxygen fractions.

The film thickness of plasma polymer films determined from ellipsometric spectra was used to evaluate the average deposition rate as the ratio between the film thickness and the deposition time. The deposition rate is an important parameter and can affect the chemical and physical properties of deposited films. The deposition rate as a function of oxygen fraction for different effective power is given in Figure 3a. We can see that the deposition ratio may vary within a wide range from 11 to 200 nm/min depending on the oxygen fraction and the effective power. For the lowest power (0.1 W), the deposition rate was approximately independent of the oxygen fraction. However, for higher power (0.5–10 W), there is a decreasing deposition rate with increasing oxygen fraction as the TVS monomer concentration decreased. The highest difference was evaluated for an effective power of 2.5 W, for which the deposition rate dropped from 200 to 38 nm/min with increased oxygen fraction. Therefore, it is beneficial to present the growth rate of plasma polymer films dependent on the effective power for various oxygen fractions (Figure 3b). Here, we can see that the deposition rate increased up to 2.5 W and then decreased for oxygen fractions ranging from 0.00 to 0.46. This trend is typical for the change of deposition mode from monomer sufficient to monomer deficient conditions according to Inagaki [19]. Higher effective power up to 10 W cannot be used for oxygen fractions ranging from 0.33 to 0.71 due to plasma instability.

### 3.3. Surface Topography of Thin Films

**Figure 4:**
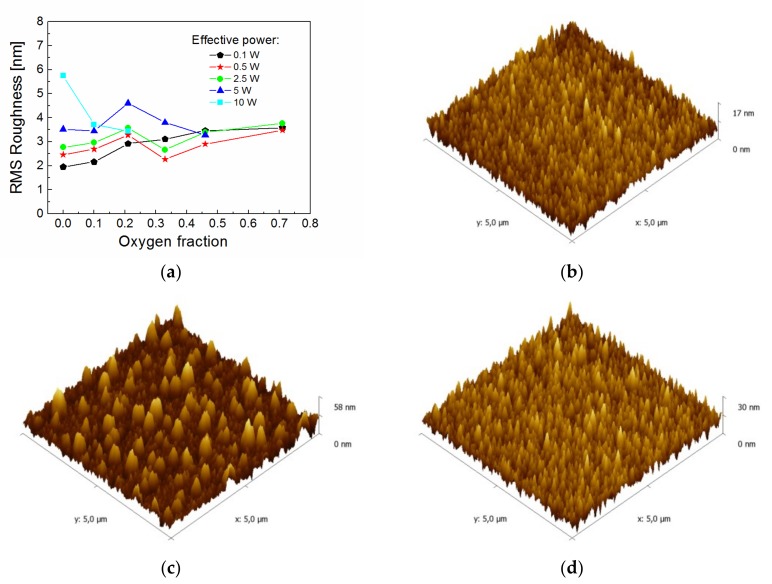
Surface topography of thin films: (**a**) RMS roughness dependent on the oxygen fraction for different effective power, (**b**) AFM image (zero oxygen fraction, 0.1 W), (**c**) AFM image (zero oxygen fraction, 10 W), (**d**) AFM image (0.71 oxygen fraction, 0.1 W).

Surface topography of deposited films was characterized by AFM measurements using a scanning area of 5 × 5 µm. Surface topography of bare silicon wafer was characterized by an RMS roughness of 1.0 nm, which was not altered after O_2_ plasma pretreatment. The RMS roughness was dependent on the oxygen fraction for the films deposited at different effective power, as shown in Figure 4a. It can be seen that the roughness of the films deposited from pure TVS monomer (zero oxygen fraction) increased from 2.0 nm (Figure 4b) to 5.8 nm (Figure 4c) with a power enhancement from 0.1 to 10 W. However, the RMS roughness was approximately the same at 3.4–3.6 nm for the highest oxygen fraction regardless of power (Figure 4d). The thickness of overlayer in the optical model used for ellipsometric spectra analysis approximately corresponded to the RMS roughness of plasma polymer films.

### 3.4. Optical Properties of Thin Films

**Figure 5:**
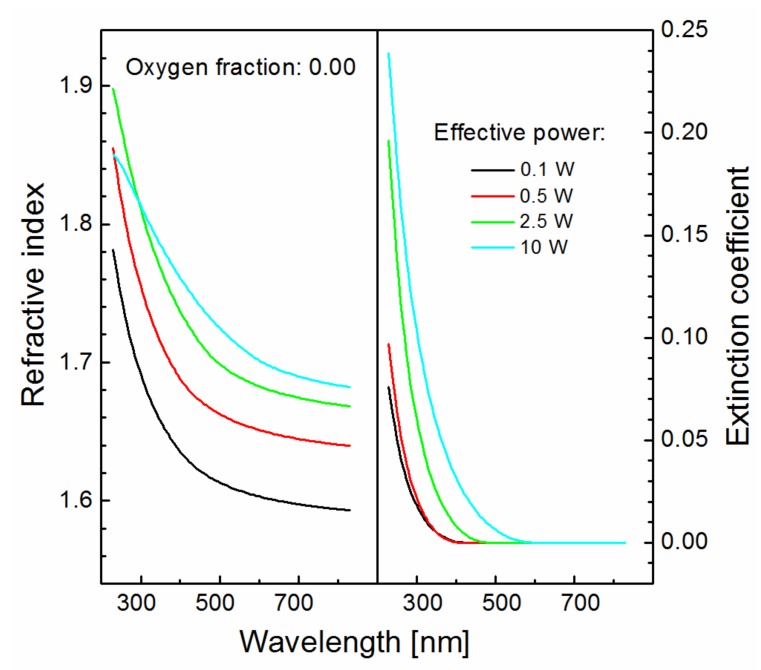
Dispersion curves for the refractive index and the extinction coefficient corresponding to the films deposited from the pure TVS monomer (zero oxygen fraction) at different effective power.

Spectroscopic ellipsometry allowed characterization of the optical properties of plasma polymer films. The dispersion curves for the refractive index and the extinction coefficient corresponding to the films deposited from pure TVS monomer (zero oxygen fraction) are given in Figure 5. The refractive index increased in the visible region while enhancing effective power. Plasma polymer films deposited at low power (0.1 and 0.5 W) were transparent in the visible region (420–830 nm) because the extinction coefficient was zero. However, the extinction coefficient gradually increased at lower wavelengths and the absorption edge (wavelength at which there is a sharp rise (discontinuity) in the extinction coefficient) was shifted towards longer wavelengths up to 605 nm for the film deposited at an effective power of 10 W. As the power increases, the plasma polymer is more crosslinked, resulting in an increase in mechanical properties, i.e., the Young’s modulus and hardness. For the same films characterized by spectroscopic ellipsometry, the Young’s modulus increased from 9.6 GPa (0.1 W) to 24.2 GPa (10 W) [6], while enhancing the effective power. Increased crosslinking of the plasma polymer resulted in its increased optical density and thus the refractive index grew with enhanced effective power, corresponding to the Clausius‒Mossotti relationship [20]:

(5) $$\frac{\varepsilon -1}{\varepsilon +2}=\frac{4\pi }{3}\rho \alpha ,$$

where ∀ is the bond polarizability and Δ is the density of the polarizable species in Equation (5).

**Figure 6:**
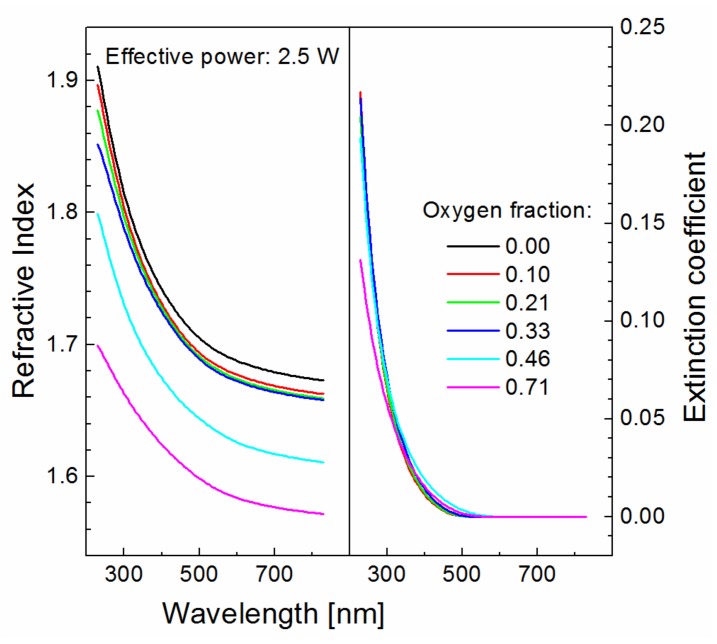
Optical properties (refractive index, extinction coefficient) of oxidized plasma polymer films deposited at an effective power of 2.5 W but various oxygen fractions.

The optical properties of oxidized plasma polymer films deposited at an effective power of 2.5 W but different oxygen fractions are shown in Figure 6. The refractive index decreased in all spectral range (230–830 nm) with an increase in the oxygen fraction. Reduction of polymer crosslinking or increase of bond strength in the plasma polymer network can be responsible for this trend. From the previous study of mechanical properties [6], we know that the Young’s modulus dropped from 19.3 to 12.2 GPa with an increased oxygen fraction for the same set of samples due to the formation of side hydroxyl and carbonyl groups, eliminating the number of carbon bonds in the plasma polymer network and resulting in reduced polymer crosslinking. We should, however, take into account the formation of a stronger Si‒O‒C network, because a weaker Si‒C bond (290 kJ/mol) was replaced by stronger Si‒O (369 kJ/mol) and C‒O (351 kJ/mol) bonds [21]. According to Phillips [22], the band gap energy, *E*_g_, for covalent materials is inversely proportional to the bond length, *d*, according to the equation:

(6) $$E_{g}\approx d^{-2.5},$$

which means that the absorption edge of the dispersion curves for the extinction coefficient must shift towards lower wavelength when increasing the oxygen fraction according to Equation (6). This trend, however, is not observed in Figure 6, and this means that only reduced crosslinking of the plasma polymer network is responsible for reducing the refractive index at increasing the oxygen fraction.

### 3.5. Correlation between Optical and Mechanical Properties of Thin Films

**Figure 7:**
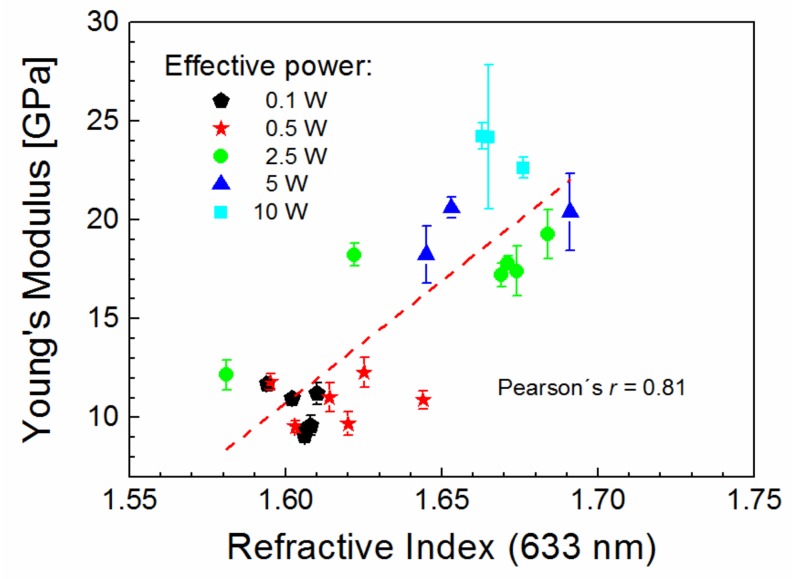
Young’s modulus correlated with the refractive index (633 nm) for oxidized plasma polymer films deposited at different effective power.

We can demonstrate that the Young’s modulus [6] can be correlated with the refractive index, represented by values at 633 nm, for plasma polymer films deposited at effective power from 0.1 to 10 W and oxygen fractions from 0.00 to 0.71 (Figure 7). Thus, it can be observed that the higher the refractive index the higher the Young’s modulus, irrespective of the power or the oxygen fraction used. The linear regression is indicated by the dashed line in Figure 7 and was used to evaluate the Pearson’s correlation coefficient, *r* [23]. Pearson’s *r* can help to measure the strength of the linear relationship between the two variables. The sequence of degree of correlation was introduced as follows [24]: 0.00–0.19 very weak, 0.20–0.39 weak, 0.40–0.59 moderate, 0.60–0.79 strong, and 0.80–1.0 very strong. Thus, Pearson’s *r* of 0.81, determined for correlation between the Young’s modulus and the refractive index, indicates a very strong positive correlation. This positive correlation confirms that both refractive index and Young’s modulus are controlled by the level of plasma polymer crosslinking. The Young’s modulus would be inversely proportional to the refractive index if the stronger bonds in the polymer network affect the optical properties of oxidized plasma polymer films [25]. This inverse trend is not indicated by the data in Figure 7.

### 3.6. Correlation between Optical and Chemical Properties of Thin Films

**Figure 8:**
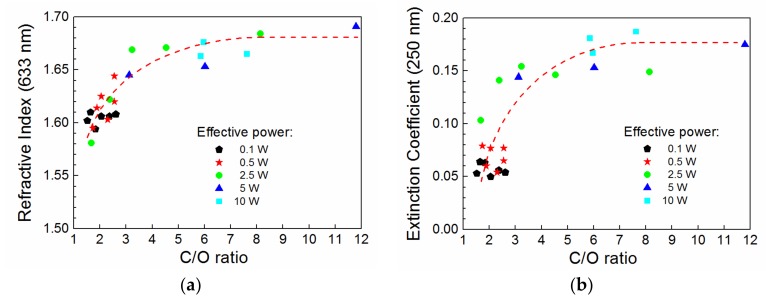
(**a**) refractive index (633 nm) and (**b**) extinction coefficient (250 nm) correlated with the C/O ratio for oxidized plasma polymer films deposited at different effective power.

It was found [6] that the C/O ratio of carbon to oxygen concentrations could be used to characterize the level of plasma polymer crosslinking and this ratio was successfully used to correlate the mechanical properties (Young’s modulus and hardness) with the chemical properties of deposited films. Because of the relation between the Young’s modulus and the refractive index (Figure 7), we can expect that the optical properties of the oxidized plasma polymer films should also correlate with their chemical properties through the C/O ratio. The refractive index (633 nm) and the extinction coefficient (250 nm) as a function of the C/O ratio are plotted in Figure 8. It is evident that the optical properties correlate well with their chemical properties for the specific deposition conditions used in this study. It is clear from Figure 8 that the plasma polymer films with the highest proportion of oxygen atoms correspond to the lowest refractive index and the extinction coefficient.

**Figure 9:**
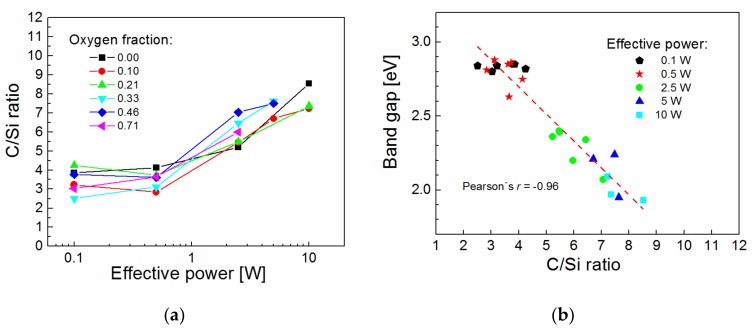
(**a**) C/Si ratio as a function of the effective power for oxidized plasma polymer films; (**b**) band gap of oxidized plasma polymer films correlated with the C/Si ratio.

The band gap is an important parameter of the plasma polymer film associated with the wavelength corresponding to the absorption edge in Figure 5 and Figure 6. This parameter does not correlate with the C/O ratio, which means that the band gap is not controlled by the level of plasma polymer crosslinking. The organic/inorganic nature of plasma polymer film may be expressed by the C/Si ratio relating the carbon and silicon concentrations. The dependence of the C/Si ratio on the effective power for plasma polymer films deposited at different oxygen fractions is given in Figure 9a. Interestingly, the organic/inorganic nature of plasma polymer films is independent of the oxygen fraction. This was explained by mass spectra of plasma species [18], from which it emerged that the oxidation process of the plasma polymer took place only on the surface of growing film but not in the bulk plasma due to oxidation of the monomer fragments. The band gap from 1.9 to 2.9 eV as a function of the chemical properties expressed by the C/Si ratio for oxidized plasma polymer films is shown in Figure 9b. The very strong negative correlation between these two variables (linear regression is indicated by the dashed line in Figure 9b) was evaluated by Pearson’s *r* of 0.96 and was independent of the oxygen fraction. The band gap values of the oxidized plasma polymer films correspond to the values for polymer-like carbon films (2–4 eV [5,26]), the value of which increased with the increased concentration of hydrogen atoms incorporated in polymer-like carbon film [27]. However, there is no correlation between the band gap and the hydrogen concentration in oxidized plasma polymer films. The increase in the C/Si ratio is accompanied by a decrease in vinyl concentration with enhanced effective power for plasma polymer films deposited from pure TVS [17]. We can therefore assume that the band gap is widened due to the higher concentration of vinyl groups in oxidized plasma polymer films at a lower C/Si ratio corresponding to a lower effective power.

## 4. Conclusions

Oxidized plasma polymer films of TVS in mixture with oxygen gas were deposited on silicon wafers at different effective power (0.1–10 W) and oxygen fractions (0–0.71) using an RF helical coupling pulsed-plasma system. Selected optical properties (refractive index, extinction coefficient, band gap) and thickness of deposited films were characterized by spectroscopic ellipsometry in the wavelength range from 230 to 830 nm. The thickness of deposited films was determined to be approximately 1 μm. The deposition rate, evaluated on the basis of film thickness and deposition time, ranged from 11 to 200 nm/min, depending on the effective power and the oxygen fraction. The RMS roughness of deposited films as measured by AFM was relatively low in the range of 2.0–5.8 nm and did not affect ellipsometric spectra analysis. Chemical analyses (RBS/ERDA, FTIR) and the previously analyzed mechanical properties of deposited films allowed the interpretation of their optical properties. The refractive index increased (1.60–1.69, λ = 633 nm) with enhanced effective power for films deposited from pure TVS due to increased crosslinking of the plasma polymer network. While the growth of the oxygen fraction decreased the refractive index (1.68–1.58, λ = 633 nm, 2.5 W) as a result of the reduction of the crosslinking of plasma polymer network. The positive correlation between the Young’s modulus and the refractive index confirmed that both parameters are controlled by the level of plasma polymer crosslinking. The influence of a stronger Si‒O‒C network on optical properties was not confirmed. It was found that the refractive index (633 nm) and the extinction coefficient (250 nm) are related to the C/O ratio, which expresses the plasma polymer crosslinking level. The band gap was related to the C/Si ratio and could be affected by the concentration of vinyl in oxidized plasma polymer films. These films with controlled mechanical, optical, and electronic properties and organic/inorganic nature can be used to construct layered nanostructures in mechanical, optical, or electronic devices.
